# Quantified Self as Epistemological Anarchism

**DOI:** 10.1007/s11406-022-00483-2

**Published:** 2022-03-04

**Authors:** Simona Chiodo

**Affiliations:** Politecnico di Milano, Milan, Italy

**Keywords:** Quantified self, Self-tracking technologies, Epistemological anarchism

## Abstract

The phenomenon of the quantified self, which is especially addressed by sociology and medical humanities, is still quite disregarded by philosophy. Yet, the philosophical issues it raises are various and meaningful, from the realm of epistemology to the realm of ethics. Moreover, it may be read as a key symptom to investigate the complex technological era in which we live, starting from the meaning of contemporary technology itself from a philosophical perspective. I shall focus on one of the epistemological issues raised by the phenomenon of the quantified self by arguing that it may be read in terms of epistemological anarchism, which also leads to other epistemological issues, such as a possibly detectable crisis of the notions of knowledge in general and science in particular as founded on the relationship between particularity and universality, as well as between reality and ideality. I shall select cases that are peculiarly representative of the founding epistemological stance I shall focus on. Yet, the reason why they deserve special attention is that they are also representative of an increasingly widespread attitude characterising not only the community of the quantified self but also, at least to some extent, anyone of us who may happen to use technologies (from apps to self-track symptoms to google to search symptoms) to try to self-diagnose.

## Introduction

The phenomenon of the quantified self, which is especially addressed by sociology and medical humanities, is still quite disregarded by philosophy (for the purpose of my article, see at least Cheney-Lippold 2011, Lupton 2012, 2016 and 2018, Dickenson 2013, Morozov 2013, Swan 2013, Nafus and Sherman 2014, Walker Rettberg 2014, Belli 2016, Neff and Nafus 2016, Selke 2016, De Groot et al. 2017, Mirza et al. 2017, Ruckenstein and Pantzar 2017, Sharon 2017, Sharon and Zandbergen 2017, Ferretti 2019, Heyen 2020). Yet, the philosophical issues it raises are various and meaningful, from the realm of epistemology to the realm of ethics. Moreover, it may be read as a key symptom to investigate the complex technological era in which we live, starting from the meaning of contemporary technology itself from a philosophical perspective.

In what follows, I shall focus on one of the epistemological issues raised by the phenomenon of the quantified self by arguing that it may be read in terms of epistemological anarchism (see especially Sect. 3, in which I shall integrate its etymological and philosophical meanings), which also leads to other epistemological issues, such as a possibly detectable crisis of the notions of knowledge in general and science in particular as founded on the relationship between particularity and universality, as well as between reality and ideality. More precisely, I shall argue that the phenomenon of the quantified self may be read in terms of epistemological anarchism, first, by analysing its definition (Sect. 2), second, by reading a first case in point, i.e. quantifying one’s health, from an epistemological perspective (Sect. 3), third, by reading a second case in point, i.e. quantifying one’s happiness, from an epistemological perspective (Sect. 4) and, fourth, by reflecting upon the move from reading the quantified self as epistemological anarchism to reading other analogous technological phenomena as epistemological anarchism (Sect. 5). Finally, I shall propose clues to further philosophical research (Sect. 6).

As a philosopher, I shall not use an ethnographic method, but I shall rely on two sources to analyse from an epistemological perspective: first, primary and secondary literature on the phenomenon of the quantified self (from what its founders write to what its scholars write) and, second, testimonies of self-trackers published in its official website^1^ (directed by Gary Wolf, who, with Kevin Kelly, defined the phenomenon of the quantified self in “Wired”, the magazine they coedited, in 2007).

## Defining the quantified self

Wolf and Kelly defined the quantified self as “self-knowledge through numbers”^2^, in that, according to them, the best way to know one’s self is given by the numbers displayed by the wearable technologies that measure one’s bodily and mental activities, starting from the increasing numbers of apps of one’s smartphone. In the last years, the words “quantified self” have been officially used not only to name the community of self-trackers who pursue the objective of quantifying their selves through the numbers displayed by their wearable technologies but also to name an academic research institute, i.e. the Quantified Self Institute in Groningen, funded by the Hanze University of Applied Science with the support of the Quantified Self Labs in San Francisco^3^.

According to Wolf, speaking of the quantified self means speaking of the move from uncertainty to certainty, i.e. what he defines as actual knowledge. Without self-tracking technologies, we “steer by guesswork. We go with our gut” (Wolf 2010, no page number also in the following quotes), and we end up making “errors of fact and errors of judgment” (Wolf 2010). Conversely, self-trackers move from seeking “a truth buried at a deeper level” (Wolf 2010) to “using numbers” (Wolf 2010), which “are making their way into the smallest crevices of our lives” (Wolf 2009)^4^: “if you want to replace the vagaries of intuition with something more reliable, you first need to gather data. Once you know the facts, you can live by them” (Wolf 2010). The first epistemological issue raised by Wolf’s words is a kind of hypertrophisation of *logos*, according to which what can provide us with actual knowledge is a restricted form of rationality coinciding with “computation, reckoning”, i.e. the literal meaning of *logos*^5^ (see at least Porter 1995). Wolf seems to exclude from actual knowledge both what we have been defining as *je ne sais quoi* since the eighteenth century and other forms of rationality, starting from what we have been defining as wisdom from the ancient Greek *metis* to Nozick’s inspiring words (see Nozick 1989: 269). In what follows, I shall not specifically focus on the issue of hypertrophisation of *logos*, which I addressed elsewhere (see Chiodo 2020a and 2020b). Conversely, I shall assume, at least to some extent, that defining the quantified self as “self-knowledge through numbers” (which is also the title of the homepage of the quantified self website) is one of the most remarkable symptoms of the increasing hypertrophisation of *logos* characterising Western culture. And I shall specifically focus on another epistemological issue, i.e. epistemological anarchism, that outwardly does not pair with hypertrophisation of *logos* – yet, as I shall argue, the two epistemological phenomena are correlated (and the quantified self is a case in point that can prove it): epistemological anarchism pairs with hypertrophisation of *logos*.

## First Case in Point: Quantifying One’s Health

In the quantified self website, the section “Show & Tell”^6^ collect hundreds of self-trackers’ presentations (videos and transcripts). Most of the self-trackers’ activities may be unquestionably useful for at least the following reason: self-tracking means paying attention to something specific, and paying attention to something specific means, at least potentially, putting oneself in the position to increase one’s awareness (as when we used to keep a diary). It is no coincidence that most of the self-trackers focus on specific health issues of which they need to increase their awareness. The list of the presentations collected in the section “Show & Tell” shows the following clusters, in which heath is pervasive: “chronic condition, cognition, diet & weight loss, environment, food tracking, genome & microbiome, heart rate & cardiovascular, location, media, metabolism, money, mood & emotion, other, ovulatory cycle & pregnancy, productivity, sleep, social life & social media, sports & fitness, stress”^7^. Yet, it is necessary to question the self-trackers’ founding epistemological stance to understand what kind of awareness the quantified self means.

I select two presentations (from several tens of presentations) as peculiarly representative of the founding epistemological stance I shall focus on (but I shall also refer to other presentations, even if I shall not analyse them in detail).

The title of the first presentation is *Where there’s data there’s hope*^8^. The self-tracker is a professor of computer science and engineering who self-diagnosed Crohn’s disease before his doctor. Thus, he became a point of reference for the community of self-trackers. He started to self-track aiming at “getting healthier”^9^. He used self-tracking technologies such as Fitbit. At some point, he wanted to take a blood test to obtain specific information. Yet, his doctor told him that there was “no insurance code for that because it’s preventative”. Thus, he resorted to the company Your Future Health^10^: “we now know it’s something like 25% of all blood tests in the US are not associated with hospitals or doctors. So I said, wow this is cool. What else can I learn?”. He learned that companies can do almost anything one can pay. Blood test after blood test, he realised to suffer from an increasing inflammation, and his question became the following: “what in the world could be causing this inflammation inside of me[?]”. He answered to his question as follows: “so because I had got out of the doctor thinking and hospital thinking and into the web thinking, while I was at yourfuturehealth.com, [I asked] what other tests could I do”. He took several tests, and concluded to suffer from inflammatory bowel disease (IBD), of which his doctor did not think (which made the former attack the latter: “my doctor who had done the colonoscopy had said you don’t have IBD […]. You know I’ve done your colonoscopy, I’ve been inside and you don’t have it. And I said […] you must be doing these all day long. He said, yeah I do, I do dozens of these a day. I said, so that’s why you don’t have time to read the scientific literature”). Finally, after having resorted to the company 23andMe^11^, he concluded to suffer from Crohn’s disease.

The story shows both a positive attitude (characterised by intellectual curiosity and tenacity) and a positive ending (finally, he could improve his health). But let us move from the reporter’s perspective to the philosopher’s perspective, according to which at least three epistemological issues deserve attention. I specify that the reason why they deserve attention is that they are representative of an increasingly widespread attitude that can be understood as founded on quite a precise epistemological stance characterising not only the community of self-trackers but also, at least to some extent, anyone of us who may happen to use technologies (from apps to self-track symptoms to google to search symptoms) to try to self-diagnose. The three epistemological issues are the following:

1. the move from feelings to numbers – which I shall try to read as hypertrophisation of *logos*;
2. the move from experts’ expertise to self-expertise – which I shall try to read as epistemological anarchism;
3. the move from relationality, including recognition of counterparts and sense of community, to individualism, and even exceptionalism – which I shall try to read, again, as epistemological anarchism.

As for the first epistemological issue, i.e. the move from feelings to numbers, we should address the following words: “don’t trust the diet book, actually measure”. More precisely, “this idea that you can feel what’s going on inside of you […] is just so epistemologically false; you just can’t do it. And so as you’ll see, there are things that are completely out of whack inside me and I would never know that by ‘feeling’, which is the first thing your doctor asks you when you come in the office. […] So what I’ve learned by this experience over 10 to 12 years is […] [that] when you go into the doctor’s office and he says how do you feel, this is next to useless. What you want to say is what are your numbers […]. And if that’s what the doctor looks at […] then you can have a useful conversation. So my endpoint where I’ve come to is where there’s data, where you can actually quantify your body and get a sense of knowledge […] there’s hope”. Two logical steps are noteworthy. The first logical step is the following: numbers can give us what feelings cannot give us, i.e. knowledge. The former, which can “measure”, can lead us to “a useful conversation” in which, if “there’s data”, i.e. quantif[ication], then there is not only “knowledge” from an epistemological perspective but also “hope” from an ethical perspective. Conversely, the latter cannot give us not only “knowledge” (“I would never know that by ‘feeling’”) but also, paradoxically enough, actual feelings (“this idea that you can feel what’s going on inside of you […] is just so epistemologically false; you just can’t do it”). Needless to say, several philosophers advocate the opposite idea, according to which feelings play a key role from an epistemological perspective (see at least Morton 2010 and Brady 2013). The second logical step is the following: if it is true that numbers can give us what feelings cannot give us, i.e. knowledge, then it is also true that not only most of us but also most of our doctors are wrong. “‘[F]eeling’ […] is the first thing your doctor asks you when you come in the office […] and he says how do you feel”. But “this is next to useless”, and should be replaced by the following question: “what are your numbers[?]”. What is especially noteworthy is that the kind of opposition that emerges is not between a rational approach, i.e. scientists’ approach, and an irrational approach, i.e., at least occasionally, non-experts’ approach. Conversely, the kind of opposition that emerges is between a restricted form of rational approach, characterised exclusively by numbers, and any other form of approach, including most doctors’ typical approach, i.e. most scientists’ typical approach. Thus, speaking of the quantified self does not mean speaking of the experts’ approach winning out over the non-experts’ approach, but speaking of quantification winning out over anything other than quantification. As such, the epistemological challenge of the self-trackers’ approach is the following: what if the realm of quantification turns out not to satisfactorily cover the realm of human life, specifically what matters most in the realm of human life? In Wolf’s words, what if it turns out not to be true that “Numbers are making their way into the smallest crevices of our lives” and “are infiltrating the last redoubts of the personal”? If quantification turns out not to satisfactorily cover human life and, moreover, if the former is believed to satisfactorily cover the latter anyway, then the epistemological reliability of quantification arises as a serious issue – the epistemological reliability of quantification risks failing (which is precisely what the COVID-19 pandemic dramatically showed us: the more complex a scenario is, the more unsatisfactory data turns out to be, specifically when it comes to taking crucial political decisions. Needless to say, Kant already taught us that stopping referring to the realm of reflective judgment means stopping referring to the vastest realm of human life. See Kant 1790).

The reason why the move from feelings to numbers may be read as hypertrophisation of *logos* should be quite clear: the latter, i.e. “computation, reckoning”, is believed to be superior to the former not only when it comes to knowing in general but also when it comes to knowing feelings themselves in particular. Symptoms, and even the general feeling of feeling good, move from being felt to being measured. Moreover, if feelings happen to contradict numbers, then the latter win out over the former anyway, as the self-trackers’ experiences show. In the case of a first testimony, the self-tracker ends up questioning the presumed coincidence between his self and his quantified self^12^, and starts to wonder if there is the risk of reducing the (vaster and more complex) former to the (less vast and less complex) latter: “We (The Apps and I) had co-constructed a digital model of my self, and here I was, managing myself, it seems, by proxy. The feedback from that digital model often took precedence over how I physically felt. When I didn’t eat ‘enough’ protein I felt weaker, and when I had too much sugar I felt fatter. These were delayed reactions; a re-reading of my body from the model. I’ve yet to decide: is this model pushing me closer in contact or further away from my self and my world?” (Williams 2013: 3). The presumed coincidence between the “digital model” and “how I physically felt” is also questioned by a second testimony, in which the self-tracker writes in verse: “Yes, I did it. / On a crisp Tuesday morning / after 40 measurements a day for 1,5 years / I. Stopped. Tracking. / Why? / […] I had stopped trusting myself / letting the numbers drown out / my intuition / my instincts[.] / Each day / my self-worth was tied to the data[.] / One pound heavier this morning? / You’re fat. / 2 g too much fat ingested? / You’re out of control. / Skipped a day of running? / You’re lazy” (Carmichael 2010, no page number). First, an opposition emerges between “myself”, “my intuition”, “my instincts” and “self-worth”, on the one hand, and “numbers” and “data”, on the other hand. Second, complexity, such as the meanings of being “out of control” and being “lazy”, risks being reduced to simplism, such as “2 g too much fat ingested” as what defines and proves her being “out of control” and “Skipped a day of running” as what defines and proves her being “lazy”. Third, simplism resulting from quantification risks leading to losing the sense of the whole, which is epistemologically essential to understand the sense of particulars.

Thus, I may anticipate that hypertrophisation of *logos* pairs with epistemological anarchism for the following reason (among other reasons I shall argue after having defined epistemological anarchism): the more we consider actual knowledge as something coinciding with a restricted form of rationality as “computation, reckoning”, the more we risk not considering at all what may fall outside of “computation, reckoning”. More precisely, we risk considering as actual knowledge what is not only reduced to simplism but also deprived of the kind of meaning that only the sense of the whole can provide us with (which, as I shall argue, is one of the most typical symptoms of epistemological anarchism).

The second epistemological issue, i.e. the move from experts’ expertise to self-expertise, can make the correlation between hypertrophisation of *logos* and epistemological anarchism clearer^13^. We should add to the words we have already quoted (“I had got out of the doctor thinking and hospital thinking and into the web thinking”, which leads to attacking the former: “so that’s why you don’t have time to read the scientific literature”) the following words: “I had to self-teach myself by going to scientific literature and using […] what Wikipedia say[s]”, “So I’m self-teaching myself this as I’m taking the data. The data is telling me what to look up and then I get these scientific articles”. From an epistemological perspective, two issues are noteworthy. First, the expert, i.e. the doctor, is substituted by non-experts both in terms of relying on the self-tracker’s expertise (yet, the self-tracker is not a doctor) and in terms of relying on others’ presumed expertise (the presumed expertise of “the web”, “the scientific literature” and “Wikipedia”) without, again, the sense of the whole, since the self-tracker, not being a doctor, cannot expertly understand what “the web”, “the scientific literature” and “Wikipedia” say by giving it the kind of meaning that only the sense of the whole can provide it with. Second, self-expertise is what ultimately matters anyway: if it is true that outwardly there are counterparts (the doctor and the authors of “the web”, “the scientific literature” and “Wikipedia”), it is also true that inwardly any counterpart is neutralised, as it were, since the ultimate meaning is given by the self-tracker, i.e. the non-expert.

Other testimonies stress an analogous attitude. As for the founders of the quantified self, they define it as “personal science”^14^ (Wolf and De Groot 2020: 1), in that it “involves the deliberate choice of the individual about what questions to ask, what methods to use and what observations to make” (Wolf and De Groot 2020: 2). More precisely, they “envision a world of personal scientists” (Wolf and De Groot 2020: 4), in that, in the case of the quantified self, differently both from citizen science and from N-of-1 trials, one and the same individual is at the same time: first, the investigator (“non-professionals occupy most, if not all, of the significant roles in research […] [that] is self-directed: the subject of the research is also the primary investigator”, Wolf and De Groot 2020: 4); second, the investigated (“The selection of topics and questions […] are determined by the researcher’s personal motive alone”, Wolf and De Groot 2020: 4); third, the user of the investigation (“the discoveries are applicable directly by the person doing the research”, Wolf and De Groot 2020: 4). Finally, a kind of rejection of what is officially defined as knowledge emerges: the “idea that we can – and should – defend ourselves against the imposed generalities of official knowledge is typical of pioneering self-trackers” (Wolf 2010).

As for other self-trackers, two testimonies are especially instructive. In the first case, the self-tracker attacks the doctors by saying that “I didn’t see that [i.e. the scientific method] when I went to my doctors. They can’t help me, so I’ll try and find these methods of my own health condition”^15^. In the second case, a popular self-tracker, praised by Wolf “for being disloyal to the professional, institutional version of science, for not conforming to scientific rituals (reported in Sharon 2017: 110. See also Wolf 2010), self-prescribed, and died of coronary occlusion at the age of sixty. By self-tracking, he ended up believing that “butter makes my brain work better. […] I really benefit from this and perhaps other people would too. […] half a stick of butter a day”^16^. After his death, his mother wrote on his blog: “Most of you won’t be surprised to learn that Seth had not visited his doctor in Berkeley in many years”^17^. Scholars from sociology and medical humanities read the phenomenon described, on the one hand, in terms of rejection of experts, i.e. doctors, in particular (“patients can and should become experts on themselves with the help of these technologies. […] this will radically alter the doctor-patient relationship, shifting the balance of power from doctors as medical experts to patients as more knowledgeable than ever about their own bodies thanks to the affordances of digital technologies that allow them to generate personal health information”, Lupton 2016: 78) and, on the other hand, in terms of rejection of rules in general (the quantified self is “a means of resistance against and a remaking of dominant social norms and conventions”, Heyen 2020: 1699).

If we try to use a more strictly philosophical, specifically epistemological, perspective, then we may read the phenomenon described as epistemological anarchism. Literally, “anarchism”, as the radicalisation of “anarchy”, means radical “absence” (*an*) of something that “rules” (*archo*) – “anarchism” means radical “rulerlessness”. In the history of Western philosophy, epistemological anarchism, as an epistemological stance typically characterising contemporary culture, was claimed by Feyerabend, who described the epistemological anarchist as whoever “becomes capable of stepping outside the most fundamental categories and convictions, including those which allegedly make him human” (Feyerabend 1975: 189), since the “‘Truth’, written ‘in capital letters’, is an orphan in this world, without power and influence” (Feyerabend 1987: 102)^18^. As such, epistemological anarchists radically separate themselves from standard ways of thinking. More precisely, they radically separate themselves from the truth. But the etymological perspective is even more illuminating – epistemological anarchists’ epistemological stance is radical rulerlessness, which also means that what rules, such as standard ways of thinking and the truth, is removed.

Outwardly, self-trackers do not seem to experience radical rulerlessness at all: they seem to pursue the truth as what numbers show, and numbers, in Western culture, are even the most standard way of thinking of the truth. Yet, inwardly, what numbers show is not the truth at all – numbers seem to be used as a kind of reassuring alibi: on the one hand, self-trackers can tell themselves to pursue the truth and, yet, on the other hand, the truth is radically sabotaged, being an idiosyncratic particular that, deprived both of a general context and of the expertise that can provide it with actual meaning, ultimately means that self-trackers are perfectly ruleless. More prosaically, self-trackers can do almost whatever they want: rulerlessness is easy when, in a perfectly solitary self-referentiality deprived both of general context and of expertise, numbers can be almost whatever one wants (it is no coincidence that scholars form sociology reflect upon the self-trackers’ self-manipulation of data, see at least Lupton 2018).

The third epistemological issue, i.e. the move from relationality, including recognition of counterparts and sense of community, to individualism, and even exceptionalism, can make the reading of the quantified self as epistemological anarchism clearer. The self-tracker’s stress on “self-teaching myself” is shared by most of the self-trackers, starting from the founders of the quantified self. The quantified self website defines itself as “A framework for personal science. Self-tracking. Self-experiment. […] Single subject research”^19^, as well as being “about our own discoveries using our own data”^20^. Moreover, the quantified self is defined in terms of individual subjects (self-trackers) who individually measure (“choosing measurements that are personally relevant rather than clinically defined”, Wolf and De Groot 2020: 3–4) what they individually want (“The selection of topics and questions […] are determined by the researcher’s personal motive alone”: “highly individual, often long term personal challenges”, Wolf and De Groot 2020: 4 and 3). A further definition of the quantified self is “the gathering of personal data for and by you. […] ‘Me and my data’, that is the point”^21^, which also leads to define it as “Functionally selfish”.

Scholars talk about breaking free (“‘tracking your weight yourself and having a doctor put you on a scale are not the same’. For her, the choice to actively track herself is, as she put it, ‘liberating’”, Sharon and Zandbergen 2017: 1702) and even escaping (see Nafus and Sherman 2014). Individualism, and even exceptionalism (see Morozov 2013), correlates with at least two issues. First, and again, a radical transformation of expertise (see Collins and Evans 2002 and 2007). Second, a radical transformation of human attitude, which moves from sociality to individuality: if it is true that “the idea that what is ‘good’, ‘right’, or ‘healthy’ for one person differs for every individual is a fundamental axiom” (Sharon 2017: 109), then it is also true that, as a self-tracker said, “‘you know yourself what is good for you or what is not good for you’” (Heyen 2020: 135). And medicine also risks moving not only from doctors’ expertise to presumed self-expertise (see also Collins 2014) but also from being public to being an individual, and even idiosyncratic, matter (see at least Dickenson 2013).

From a more strictly philosophical, specifically epistemological, perspective, the reason why we may read the phenomenon described as epistemological anarchism is the following: individualism, and even exceptionalism, results from the removal of any actual otherness as an actual counterpart – conversely, the relationship between what is individual, i.e. particular, and its counterpart, i.e. what is universal, is nothing less than the epistemological cornerstone of knowledge as it has been thought of by Western culture for millennia. According to Western epistemology, starting from the ancient Greek introduction of the dimension of universality as the perfect counterpart of the dimension of particularity (see at least Berlin 1988, 1990 and 1996 and Jullien 2009,22), the meaning of P (particularity) is given by its comparison with U (universality). More precisely, the meaning of P is given by its comparison with its ideal model, which is universal, since it is obtained through the following epistemological process: first, we analyse several Ps by identifying what makes them be different from each other (which is the epistemological process of analysis); second, we abstract from several Ps by identifying what they share anyway, i.e. what is the reason why, even if they are different from each other, we define all of them by applying one and the same word, as when we apply the word “triangle” to several triangles that are different from each other (which is the epistemological process of abstraction); third, we idealise by perfecting, exclusively in our imagination, what results from abstraction, sometimes by removing something and sometimes by adding something (which is the epistemological process of idealisation). Finally, we obtain ideal models, which can guide not only our science but also our art, our law and our philosophy itself. And we use ideal models as the Us that, as Kant masterfully explained, can make us successfully “compare […], judging […] and thereby improving” (Kant 1781: A 569/B 597) the Ps. As such, ideal models “have a practical power (as regulative principles)” (Kant 1781: A 569/B 597). But “regulative principles” are precisely what the self-trackers’ founding epistemological stance removes: the self-trackers’ individualism, and even exceptionalism, leads to Ps that are not actually compared with Us at all – the self-trackers’ “fundamental axiom” according to which “what is ‘good’, ‘right’, or ‘healthy’ for one person differs for every individual” leads to think of “regulative principles” precisely as the rules from which one should “liberat[e]”, and even escape: again, the ultimate result is becoming perfectly ruleless, in a scenario characterised sometimes by ruling Us whose actual use is radically sabotaged (for instance, when they do not result at all from expert analysis, abstraction and idealisation) and sometimes by the removal itself of ruling Us (for instance, when the comparison of P with U is removed by the absolutisation of the former, which becomes self-referential).

Thus, the phenomenon we investigate may be read as epistemological anarchism not only because it questions the notion of expertise, as well as of knowledge, but also because it questions the relationship between P and U as the millennial epistemological cornerstone of knowledge as it has been thought of in Western culture. Are we starting to face a crisis of knowledge in general, as well as of science in particular, as founded on the comparison of real Ps with ideal Us? More precisely, why ideal Us as rules shared by all real Ps seem to face a crisis?

## Second Case in Point: Quantifying One’s Happiness

The second presentation I select as peculiarly representative of the founding epistemological stance I focus on is *Happsee*^23^. The self-tracker is the user of the app he created when, after having decided “to measure his happiness and mood to improve it”^24^, he also decided that other self-tracking technologies “did not meet his needs”. His app is based on smartphone sensors that, through self-tracking, collect his data to quantify his happiness.

The same three epistemological issues arise:

1. the move from feelings to numbers, i.e. hypertrophisation of *logos*;
2. the move from experts’ expertise to self-expertise, i.e. epistemological anarchism;
3. the move from relationality, including recognition of counterparts and sense of community, to individualism, and even exceptionalism, i.e., again, epistemological anarchism.

Let us try not only to review them but also to try to step forward.

We can find the first epistemological issue, i.e. the move from feelings to numbers (hypertrophisation of *logos*), in what follows. According to the self-tracker, happiness, as well as unhappiness, is not something to feel (inside), but something to know (outside): “I was in a situation where I thought I was unhappy. So I thought I was in this situation where I thought I was unhappy […]. [But] I didn’t know if I was unhappy”. That is, feeling and thinking to be unhappy, on the one hand, and knowing to be unhappy, on the other hand, are considered as divergent. More precisely, unhappiness moves from being a non-quantifiable internal truth whose existence is proved by one’s feeling and thinking to being a quantifiable external truth whose existence is proved by self-tracking technology’s knowing. The move from something internal to something external, i.e. the epistemological externalisation of unhappiness, is explicit: “So I wanted to explore this whole space of unhappiness and how to improve it. So how do we do that? I wanted to measure my happiness and mood”, which means creating a quantifying self-tracking technology, i.e. “this android app called Happsee”. The epistemological externalisation of unhappiness exemplifies the way we seem to increasingly use technology: first, we increasingly think that computational knowledge is better than non-computational knowledge (and even that non-computational knowledge is not knowledge at all); second, we increasingly think that technology’s computational knowledge is better than the human mind’s computational knowledge; third, we increasingly externalise knowledge from the human mind to technology – which means that, paradoxically enough, we increasingly stop exercising the capacity that, in Western culture, has been defining the core of human identity for millennia: the capacity to actually know, and to make decisions accordingly.

As for the second epistemological issue, i.e. the move from experts’ expertise to self-expertise (epistemological anarchism), we can find it in what follows. The self-tracker says that he does not want to use apps designed by others. Conversely, he wants to use the app he designs as tailored to his individual needs: “So I looked at a lot of measurements tools. […] Most of them did not suit my needs. They were either too complex. They asked me to fill out a bunch of surveys and I think a few of them actually made me angry, because I was like [asking myself] why am I filling out all these questions and that completely defeats the purpose of understanding whether I’m happy. […] So in the spirit of software I made my own tool”. A kind of self-referentiality emerges from the self-tracker’s words, in that he escapes both from possible comparisons with others in general and from possible comparisons with others’ expertise in particular (for instance, with scholars of happiness). And, if we address the kind of knowledge resulting from the self-tracker’s self-referentiality, we can find at least four noteworthy issues. First, he says that what he did to use his app was to “enter […] in some moods. I entered in how happy I was on a zero to ten scale”, and his app made graphs and maps also based on passive data such as geographical positions. We may note that what he did opposes what he says about the divergence between feeling and thinking, on the one hand, and knowing, on the other hand, since he actually “entered in” what he felt and thought (“how happy I was”) by simply translating it into numbers (“on a zero to ten scale”). Second, even if his knowledge is actually founded on what he felt and thought, a kind of placebo effect emerges, in that “uncertain is dropping. Stressed was in don’t know […] and my positive moods have […] been going up”. Thus, his questionable knowledge triggers his behaviours. Third, his knowledge is not only questionable but also obvious: “when I started to get less tired, I started to get a lot happier”. Yet, obviousness seems reassuring, and not suspicious at all. And he even wants to step forward: “to use machine learning to predict happiness”. Thus, his obvious knowledge also triggers his behaviours. (We can frequently find cases of obvious knowledge in the self-trackers’ presentations. Yet, and again, obviousness seems reassuring, and not suspicious at all: “So I extracted them and asked my computer well, what makes me happy and he says well […], just be active, have a good night sleep and don’t be stressed. And that’s pretty nice to know that actually what you […] think will make you happy does make you happy”^25^.) Fourth, his knowledge is suspicious not only because it is actually founded on what he thinks it is not founded on and because it is obvious but also because it is suspiciously tailored to his individual idiosyncrasies: “I’m lazy, and the one thing that gets me annoyed a little bit is […] to make a lot of entries to tell the system how happy I am. What if it could just pick that up from passive data[?]”. We may note that, again, the meaning of P (particularity) is not given by its comparison with U (universality). Conversely, the meaning of P (as his own idiosyncratic happiness) is given by its confirmation through P (as his own idiosyncratic app).

Symptoms of epistemological anarchism are especially identifiable in the radical epistemological self-referentiality characterising the process described from start to finish. Again, a perfectly solitary self-referentiality deprived both of general context and of expertise easily leads both to a kind of epistemological circularity, in that happiness as the (suspicious) output is founded on happiness as the (suspicious) input, and to a kind of epistemological surrender, in that human alertness and critical thinking seem to decrease. More precisely, the reason why human alertness and critical thinking seem to decrease is that what is self-acquired is so individually satisfactory that actual obviousness is believed to be actual knowledge: what is self-acquired, whatever it is, increases a kind of individual satisfaction that, perfectly deprived of contradicting comparisons, easily falls into substituting not only less reassuring alertness and critical thinking with more reassuring self-confidence but also less reassuring knowledge as constitutively related to expert counterparts with more reassuring obviousness as constitutively self-related. Thus, expertise itself risks being radically devalued both when, in general, it has to do with exercising human alertness and critical thinking and when, in particular, it has to do with exercising human expertise (for instance, psychologists’ expertise in happiness). Moreover, and again, what founds expertise itself risks being radically sabotaged, i.e. the ideal model as the most powerful epistemological tool introduced by Western culture to know what is particular: knowing what is particular moves from referring it to what is universal to referring it to what is not only particular but also self-referentially idiosyncratic.

As for the third epistemological issue, i.e. the move from relationality, including recognition of counterparts and sense of community, to individualism, and even exceptionalism (epistemological anarchism), we can find it both in what we have already seen and in what follows. The self-tracker specifies that to “suit my needs” especially means “to maximize information gathered while minimizing the information I actually had to type in because I’m lazy”. Again, being tailored to individual needs seems to mean being tailored to individual idiosyncrasies, no matter if they may interfere with the quality of knowledge. A kind of idiosyncratic self-referentiality seems to characterise not only the self-tracker’s knowledge of his self but also the self-tracker’s perception of his self. In the first case, as we have already seen, the knowledge of his self becomes self-referential through the removal of the comparison between particularity and universality, i.e. ideal models. In the second case, the perception of his self becomes self-referential through the removal of the comparison between him as a particular individual and others as other particular individuals (who are potentially contradicting). As a sociologist puts it, also “public presentations of self-tracking research at meetups or conferences make hardly any references to other self-trackers and their activities” (Heyen 2020: 133). Moreover, if “the self-tracker can only reproduce a research study *on himself or herself*, because here the researcher and the test subject are one and the same person” (Heyen 2020: 131), then “objectivity in the sense of intersubjective verifiability or reproducibility of the measurement plays almost no role in the meetup discussions” (Heyen 2020: 131). From a philosophical perspective, the perception of the self moves from being shaped and assessed through comparisons with other individuals to being shaped and assessed through self-comparisons, i.e. comparisons between the self-tracker’s own data over time. Needless to say, idiosyncratic self-referentiality easily leads to epistemological oversimplification, in a kind of self-reinforcing vicious circle: for instance, the exceedingly complex meaning of happiness is deprived of the infinite intake provided both by ideal models and by other real individuals, and ends up coinciding with what happiness is exclusively for the self-tracker and exclusively on the basis of the self-tracker’s own data over time. Again, rulerlessness characterises the self-tracker’s founding epistemological stance: even if, outwardly, rules are not only present but also reinforced by moving from feelings to numbers, inwardly, numbers’ epistemological reliability is radically sabotaged by an extreme self-referentiality in which the meaning provided by a potentially contradicting general context is substituted by individualism, and even exceptionalism, and the meaning provided by a potentially contradicting expertise is substituted by non-expert self-expertise – and, again, epistemological anarchism arises as the kind of rulerlessness that seems to be actually implied by most of our ways of using our technologies, from when we self-diagnose by googling our symptoms to when we make complex decisions by making algorithms process data and by doing exactly what they tell us to do.

## From the Quantified Self’s Epistemological Anarchism to Our Selves’ Epistemological Anarchism

At times, self-trackers end up saying that their selves cannot be quantified, even after 28 years of self-tracking, which is a part of the title of the presentation *28 years of tracking, but what have I learned?*^26^: “So I have learned that I’m not in fact an engineering problem of calories in and calories out. There is a lot more complex and subtle interactions going on that keep me constantly adjusting. What worked the first time didn’t work the second time”. The self-tracker realises that her self goes exceedingly far beyond her quantified self. But her realisation, and even her possible decision to stop self-tracking, does not mean that she will not risk falling into epistemological anarchism anyway: the quantified self is the extreme point of a line on which most of us can position ourselves without being necessarily regular self-trackers.

Whenever, for instance, we self-diagnose by googling our symptoms, we may fall into epistemological anarchism by removing what “rules” (*archo*) as a form of heteronomy, i.e. as doctors who can rule us as experts. Moreover, whenever, for instance, we make complex decisions by making algorithms process data and by doing exactly what they tell us to do, we may fall into epistemological anarchism by removing what “rules” (*archo*) as a form of autonomy, i.e. as our epistemological and ethical reasons that can rule us as what was identified by Kant as nothing less than the core of human identity (and dignity). Paradoxically enough, even if we, as Western humans, come from a cultural tradition according to which the notion of human identity (and dignity) has been founded on the notion of autonomy, we seem to increasingly use technology to shift autonomy from ourselves to technology, as several technologies’ definitions show, such as autonomous vehicles, autonomous systems, autonomous software, autonomous devices, autonomous applications, autonomous silicon, autonomous machines, autonomous equipment, autonomous drones, autonomous weapons, autonomous robots, autonomous agents, autonomous workloads and so forth. More precisely, we seem to increasingly trade (human) autonomy for (technological) automation – and trading (human) autonomy for (technological) automation is one of the most anarchic moves we may make.

From an etymological perspective, whenever we trade, for instance, our autonomous decisions funded on our epistemological and ethical reasons for algorithms’ automated decisions, we trade something that, being “autonomous”, is a “self-given law” with something that, being “automated”, is not a “law” at all – and radically removing the “law” means taking the first step toward anarchism. More precisely, being “automated” means what the ancient Greek verb *automatizo* means: to “act of oneself, act offhand or unadvisedly”^27^, “to be done spontaneously or at random”, “haphazard”, to “introduce the agency of chance”, “of things, [to] happen of themselves, casually”, “to be self-produced” and, “of natural agencies, [to] act spontaneously”. (And, interestingly enough, the ancient Greek noun *automaton* means “accident”^28^, the ancient Greek noun *automatismos* means “that which happens of itself, chance”^29^, and the ancient Greek noun *Automatia* is “the goddess of chance”^30^, defined by Smith as a “surname of Tyche or Fortuna, which seems to characterize her as the goddess who manages things according to her own will, without any regard to the merit of man”^31^.) Thus, whenever we trade our autonomous decisions funded on our epistemological and ethical reasons for algorithms’ automated decisions, we trade autonomy for the first step toward anarchism, in that we substitute “self-given laws” not only with the “absence” (*an*) of “laws”, i.e. something that “rules” (*archo*), but also with the presence of the kind of “random[ness]” that being “automated” means: whatever algorithms tell us to do, their reasons are, at least to some extent, inscrutable to us not only because of technical reasons but also because of their constitutive separateness from the kind of individual responsibility that makes what is “self-given” even more “self-given”, as it were. When we make the wrong decision through (human) autonomy, we are likely to bear its burden forever (as most of Western literary masterpieces show us, from Aeschylus to Shakespeare to Pirandello). Conversely, when we make the wrong decision through (technological) automation, we are likely to bear its burden for hours, if any. If it is true that (human) autonomy and (technological) automation can promisingly work together, it is also true that we seem to increasingly substitute the former’s action with the latter’s action (I myself happened to be told that I should not have to take a potentially crucial test not by doctors, but by a letter literally saying that, according to the predictive algorithm they used, I should not have to take it. And, moving from my experience to global phenomena, the management of the COVID-19 pandemic, for instance, may be considered as a clear example of the increasing substitution of human autonomous decisions made by politicians with technological automated decisions made by data: it is no coincidence that one of the most dramatic crises the COVID-19 pandemic showed us is the crisis of politics, which is precisely one of the most typical realms in which human autonomy, defined as individual responsibility both for making complex decisions and for bearing their burdens, should be exercised).

From a philosophical perspective, the quantified self can help us understand a phenomenon that seems to characterise our technological era *tout court* – and, again, the reason is that the quantified self takes to extremes a founding epistemological stance that goes exceedingly far beyond it, and which may be read as epistemological anarchism.

First, as we have already seen, we can find epistemological anarchism in the crisis of expertise. Moving from the quantified self to more general phenomena, we can find the crisis of expertise whenever we self-diagnose by googling our symptoms, not only because we substitute doctors’ expertise with our non-expert self-expertise but also because we substitute the realm of “laws”, where the experts’ identities are clearly exposed and responsible for what they say, with the realm of “random[ness]”, where the experts’ identities are not clearly exposed and responsible for what they say. And we can find the crisis of expertise whenever we make complex decisions by making algorithms process data and by doing exactly what they tell us to do, not only because we increasingly lose our expertise in making complex decisions by increasingly externalising it from ourselves to technology (which means by increasingly atrophying it) but also because we substitute human capacities that can work on non-computable data with technological capacities that can work on nothing but computable data. Finally, and more generally, we can find the crisis of expertise whenever we haunt social media with millions of non-expert judgements on whatever, ending up being even the worst kind of epistemological anarchist, i.e. the epistemological apathetic.

Second, as we have already seen, we can find epistemological anarchism in the crisis of knowledge in general, as well as of science in particular, as founded on the relationship between particularity and universality, as well as between reality and ideality. Moving from the quantified self to more general phenomena, we can find the crisis of the relationship between real particularity and ideal universality, i.e. the ideal model, whenever we self-diagnose by googling our symptoms, in that we rely on the relationship between a first idiosyncratic particular (our symptoms) and a second idiosyncratic particular (Google’s algorithm, which today happens to display, for inscrutable reasons, the explanation X as the most relevant and tomorrow happens to display, for inscrutable reasons, the explanation Y as the most relevant. Yet, both X and Y cannot provide us with actual knowledge, since no expert assessed the quality of the relationship between them and our symptoms). And we can find the crisis of the ideal model whenever we make complex decisions by making algorithms process data and by doing exactly what they tell us to do, in that we rely on a kind of abstraction that, being technological, may happen not to cover at all what the human mind’s abstraction can cover, even if on a subconscious level (which is what may happen whenever we make up our minds, ending up making the best decisions, after having slept on them. We cannot do without asking ourselves: from what non-computable, and even subconscious, data did our minds abstract? And what data could not have been covered by algorithms? That is, is there something that cannot be data for algorithms, but that can be data for the human mind?). Finally, and more generally, we can find the crisis of the ideal model whenever we consider ourselves as the exception to the rule – and the rule, exception after exception, ends up dissolving in rulerlessness, i.e. epistemological anarchism. The quantified self takes to extremes a founding epistemological stance that risks increasingly characterising, for instance, our notion of health *tout court*, starting from personalised medicine (which may be a great resource, but which makes us question the ideal model’s future: what is the destiny of universality if particularity rejects its ruling?).

Third, we can also find epistemological anarchism in a deeper meaning of the crisis of the ideal model. Several scholars who correlate the quantified self, as well as analogous phenomena, with neoliberalism make us see a kind of chain resulting from the following rings: first, neoliberalism demanding individuals to equal their ideal models; second, individuals trying to be at least “their ‘best selves’” (Lupton 2016: 48) even by regularly self-tracking; third, several individuals ending up having breakdowns; fourth, ideal models ending up being rejected by several individuals. Lupton remarks, from a sociological perspective, that being “their ‘best selves’”, i.e. “behaving as responsible citizens, engaged in self-care […], engaging in self-optimisation or enhancement is even demanded of people” (Lupton 2016: 48) in several competitive contexts. Thus, as a columnist for *The New York Times* says, “he engaged in self-tracking because he believed that ‘[y]ou want to be your best self’” (Lupton 2016: 65). More generally, if one thinks of “ideal selfhood” (Lupton 2016: 50) as something to make real, and not as a regulative ideal, then “Part of this practice requires self-monitoring” (Lupton 2016: 49) by regularly self-tracking as rigorously as possible. From an epistemological perspective, the vicious circle may be read as follows:

1. ideal models move from being considered as regulative ideals to being considered as something to make real;
2. individuals strive for making ideal models real. Yet, as Plato already taught us, making something ideal real is epistemologically and ontologically impossible (see Plat. *Resp*. 596 a-b);
3. thus, a desperate striving easily falls into a radical attempt to reduce complexity to something more reassuring. And what may be more reassuring, for Western humans, than a kind of technological computation that (outwardly) reduces complexity to simplism by (outwardly) quantifying even something ideal?
4. Yet, both Western humans and their ideal models do not seem reducible to “an engineering problem”.

But we keep trying to use technology to reduce complexity to a kind of reassuring simplism that sometimes, as we have already seen, seems to increase our self-confidence even through obviousness. Yet, and again, obviousness is nothing less than a further emergence of epistemological anarchism: whenever we substitute complexity with obviousness we deprive (*an*) of what rules (*archo*) not only knowledge itself (in that we move from a notion of knowledge as S is P, which adds something, to a notion of knowledge as S is S, which adds nothing) but also ourselves (in that we move from the presence of a potentially contradicting P to its radical absence) – and we become perfectly ruleless.

## Conclusions

The founders of the quantified self also add that it introduces a novel notion of self: the self becomes an exoself through the increasing number of wearable technologies we use (see Kelly 2012, as well as Swan 2013 and Sharon 2017). From a philosophical perspective, the notion of exoself may be read as a further kind of externalisation, being not only an epistemological externalisation, as we have already seen, but also an ontological externalisation: speaking of exoself means externalising not only knowledge, by moving it from the human mind to technology, but also the being, by moving it from the human body to technology – may we read the exoself as a further step toward anarchism, in that, through an even more radical externalisation, we deprive (*an*) ourselves not only of a ruling (*archo*) epistemology we are irreducibly responsible for but also of a ruling (*archo*) ontology we are irreducibly responsible for?

Even if the question requires to be addressed in a specific publication, I may conclude by saying at least what follows. Actually, we seem to use technology to try to make it increasingly bear our burdens for us – more precisely, to try to make technology increasingly bear any kind of human burden, from epistemological burdens to ontological burdens. And we may even think of a further kind of burdens, which has to do with ideality considered even from a theological perspective. Actually, we seem to use technology not only to make it obtain human prerogatives and burdens (as we have already seen especially through the notion of autonomy’s move from the human realm to the technological realm) but also to make it obtain divine prerogatives and burdens. If we ask ourselves in what realm we increasingly happen to use typically theological notions such as omnipresence, omniscience, omnipotence and inscrutability, then we should answer that we increasingly happen to use them to define technology – a kind of technology that, from when we use it to google our symptoms to when we use it to make algorithms make decisions for us, becomes more and more omnipresent, omniscient, omnipotent and inscrutable. Yet, it is not transcendent at all. Conversely, technology is totally immanent, being a totally immanent human creation – and substituting a transcendent divine with a totally immanent divine may be thought of as the most anarchic move we may make in our history.

## Footnotes

1 See https://quantifiedself.com/.

2 As the quantified self website reports, see https://quantifiedself.com/ (accessed in April 2021).

3 See https://qsinstitute.com/.

4 More precisely, “numbers are infiltrating the last redoubts of the personal. Sleep, exercise, sex, food, mood, location, alertness, productivity, even spiritual well-being are being tracked and measured” (Wolf 2010).

5 See http://www.perseus.tufts.edu/hopper/text?doc=Perseus%3Atext%3A1999.04.0057%3Aentry%3Dlo%2Fgos (accessed in April 2021).

6 See https://quantifiedself.com/show-and-tell/.

7 See https://quantifiedself.com/show-and-tell/ (accessed in April 2021).

8 See https://quantifiedself.com/show-and-tell/?project=390.

9 See https://quantifiedself.com/show-and-tell/?project=390 also for the following quotes (accessed in April 2021).

10 See https://www.yourfuturehealth.com/.

11 See https://www.23andme.com/.

12 It is no coincidence that “‘I am what I track’ is a statement that is commonly heard at QS [the quantified self] gatherings” (Sharon 2017: 115).

13 Even though there are cases in which moving from experts’ expertise to self-expertise may be considered as promising from an epistemological perspective (see Levy and Alfano 2020), in what follows, I shall especially argue the reasons why the case of the quantified self may be read as one of the symptoms of epistemological anarchism (even though, in Sect. 3, I myself also acknowledge a positive attitude at work, starting with intellectual curiosity and tenacity).

14 The notion of personal science comes from Polanyi’s stress on subjectivity in scientific practice, from discovery to validation (see Polanyi 1958), as well as from other scholars’ reflections upon the relationship between knower and knowledge, with a recent stress on the quantified self (see especially Heyen 2020 but also Martin and Brouwer 1993 and De Groot et al. 2017).

15 See https://quantifiedself.com/show-and-tell/?project=563 (accessed in April 2021).

16 See https://quantifiedself.com/show-and-tell/?project=206 (accessed in April 2021).

17 See https://sethroberts.net/category/general/ (accessed in April 2021).

18 Feyerabend’s reference to Dadaism further clarifies: “like the Dadaist, […] he [the epistemological anarchist] ‘not only has no programme, [he is] against all programmes’” (Feyerabend 1975: 189). He especially refers to Tzara’s *Dada manifesto*, according to which “I am against systems, the most acceptable system is the one of not having any system, on principle” (Tzara 2001: 299). Yet, “I am also against principles” (Tzara 2001: 300): “There is no final Truth” (Tzara 2001: 298) and there is no ideal (“If I shout: IDEAL, IDEAL, IDEAL, KNOWLEDGE, KNOWLEDGE, KNOWLEDGE, BOOMBOOM, BOOMBOOM, BOOMBOOM, I have put down rather exactly the progress, the laws, morality, and all the other lovely qualities that various very intelligent people have discussed in so many books, just in order to say finally that each man has danced anyway according to his own personal boomboom, and that he is right in his boomboom”, Tzara 2001: 302).

19 See https://quantifiedself.com/ (accessed in April 2021).

20 See https://quantifiedself.com/blog/ (accessed in April 2021).

21 See http://qsinstitute.com/quantified-self-quantified-us-quantified-other/ also for the following quote (accessed in April 2021).

22 I myself worked on the issue, specifically Western epistemology as underpinned by the introduction of the dimension of ideality (and universality) as the perfect counterpart of the dimension of reality (and particularity), especially in Chiodo 2016.

23 See https://quantifiedself.com/show-and-tell/?project=723.

24 See https://quantifiedself.com/show-and-tell/?project=723 also for the following quotes (accessed in April 2021).

25 See https://quantifiedself.com/show-and-tell/?project=847.

26 See https://quantifiedself.com/show-and-tell/?project=815 also for the following quote (accessed in April 2021).

27 As reported by the Liddell-Scott-Jones Greek-English Lexicon. See http://stephanus.tlg.uci.edu/lsj/#eid=18225 also for the following quotes (accessed in May 2021).

28 See http://stephanus.tlg.uci.edu/lsj/#eid=18228 (accessed in May 2021).

29 See http://stephanus.tlg.uci.edu/lsj/#eid=18226 (accessed in May 2021).

30 See http://stephanus.tlg.uci.edu/lsj/#eid=18224 (accessed in May 2021). See also Murray 1833: 577.

31 Quote from Smith 1867, see https://quod.lib.umich.edu/m/moa/acl3129.0001.001/462?page=root;rgn=full+text;size=100;view=image;q1=auto (accessed in May 2021).
